# Molecular Characterization and Tandem Mass Spectrometry of the Lectin Extracted from the Seeds of *Dioclea sclerocarpa* Ducke

**DOI:** 10.3390/molecules16119077

**Published:** 2011-10-28

**Authors:** Jorge Luis Almeida Correia, Antônia Sâmia Fernandes do Nascimento, João Batista Cajazeiras, Ana Cláudia Silva Gondim, Ronniery Ilario Pereira, Bruno Lopes de Sousa, André Luiz Coelho da Silva, Wanius Garcia, Edson Holanda Teixeira, Kyria Santiago do Nascimento, Bruno Anderson Matias da Rocha, Celso Shiniti Nagano, Alexandre Holanda Sampaio, Benildo Sousa Cavada

**Affiliations:** 1 Laboratório de Moléculas Biologicamente Ativas (Biomol-Lab), Departamento de Bioquímica e Biologia Molecular, Universidade Federal do Ceará, Av. Humberto Monte s/n, Bloco 907, Lab. 1075, Campus do Pici, Fortaleza-CE, 60440-970, Brazil; 2 Laboratório de Biotecnologia Molecular (LabBMol), Departamento de Bioquímica e Biologia Molecular, Universidade Federal do Ceará, Av. Humberto Monte s/n, Bloco 907, Lab. 1090, Campus do Pici, Fortaleza-CE, 60440-970, Brazil; 3 Centro de Ciências Naturais e Humanas, Universidade Federal do ABC, Santo Andre-SP, 09210-170, Brazil; 4 Laboratório de Imunologia e Bioquímica de Sobral (LIBS), Faculdade de Medicina, Universidade Federal do Ceará, Sobral-CE, 62042-280, Brazil; 5 Laboratório de Espectrometria de Massa Aplicado a Proteínas (LEMAP/Biomol-Lab), Departamento de Engenharia de Pesca, Universidade Federal do Ceará, Av. Humberto Monte s/n, Bloco 825, Campus do Pici, Fortaleza-CE, 60440-970, Brazil

**Keywords:** plant lectin, diocleinae, *Dioclea sclerocarpa*

## Abstract

Lectin from the seeds of *Dioclea sclerocarpa* (DSL) was purified in a single step by affinity chromatography on a Sephadex G-50 column. The primary sequence, as determined by tandem mass spectrometry, revealed a protein with 237 amino acids and 81% of identity with ConA. DSL has a molecular mass of 25,606 Da. The β and γ chains weigh 12,873 Da and 12,752 Da, respectively. DSL hemagglutinated rabbit erythrocytes (both native and treated with proteolytic enzymes), showing stability even after one hour of exposure to a specific pH range. The hemagglutinating activity of DSL was optimal between pH 6.0 and 8.0, but was inhibited after incubation with D-galactose and D-glucose. The pure protein possesses a molecular mass of 25 kDa by SDS-PAGE and 25,606 Da by mass spectrometry. The secondary structure content was estimated using the software SELCON3. The results indicate that b-sheet secondary structures are predominant in DSL (approximately 42.3% antiparallel b-sheet and 6.7% parallel b-sheet). In addition to the b-sheet, the predicted secondary structure of DSL features 4.1% a-helices, 15.8% turns and 31.3% other contributions. Upon thermal denaturation, evaluated by measuring changes in ellipticity at 218 nm induced by a temperature increase from 20 °C to 98 °C, DSL displayed cooperative sigmoidal behavior with transition midpoint at 84 °C and permitted the observation of two-state model (native and denatured).

## 1. Introduction

Lectins are a class of carbohydrate-binding proteins of non-immune origin organized into closely structurally related families [1]. These proteins are ubiquitously distributed and posses at least one domain able to reversibly and specifically recognize carbohydrate moieties without altering its covalent structure [1,2,3,4]. Lectins have been studied extensively in recent years because of their usefulness as molecular tools in investigations of cell surface composition/changes, growth, differentiation, and aggregation, as well as in studies of several pathological mechanisms and in the isolation and characterization of glycoconjugates [5,6].

Although widely distributed in Nature, the plant-isolated lectins are the most extensively studied, being found in roots, bulbs, bark, and leaves. Lectins isolated from leguminous plants, such as peas and beans, are the most extensively investigated. Despite differences in carbohydrate-binding specificity and quaternary structures, legume lectins present high sequential identity, and the monomers share the same tertiary structure, which is characterized by an essentially conserved “jelly roll” motif [7].

Diocleinae lectins are a well studied group of closely related lectins among the leguminous group. Different biological effects associated to these proteins have been described such as histamine release from rat peritoneal mast cells [8] and anti- and pro-oedematogenic effects [9,10]. Minor differences in the ratios of dimeric and tetrameric forms in the lectins, together with differences in the relative orientations of the carbohydrate-binding sites in the quaternary structures, have been hypothesized to contribute to the differences in biological activities exhibited by Diocleinae lectins [11]. These interesting properties make Diocleinae lectins valuable biotechnological tools, being consider of great interest the characterization and sequence analysis of different lectins belonging to this subtribe. Furthermore, Diocleinae lectins provide an excellent system to study the dramatic effects of minor structural differences on functional properties in proteins [12].

In the present study, we isolated a glucose/mannose-binding lectin (DSL) from the seeds of *Dioclea sclerocarpa* Ducke, a woody vine of the Diocleinae subtribe found in Northeastern Brazil. The purified lectin was physicochemically characterized, submitted to primary structure analysis by mass spectrometry and compared to other Diocleinae lectins.

## 2. Results and Discussion

The crude extract of *D. sclerocarpa* seeds induced strong agglutination activity with both native and trypsinized rabbit erythrocytes. The carbohydrate binding speciﬁcity of the crude extract was found to be against glucose and mannose, with mannose as the most potent (minimum concentration: 20 mM), indicating a hemagglutinating profile similar to that of other Diocleinae lectins [13,14,15,16,17,18,19].

**Figure 1:**
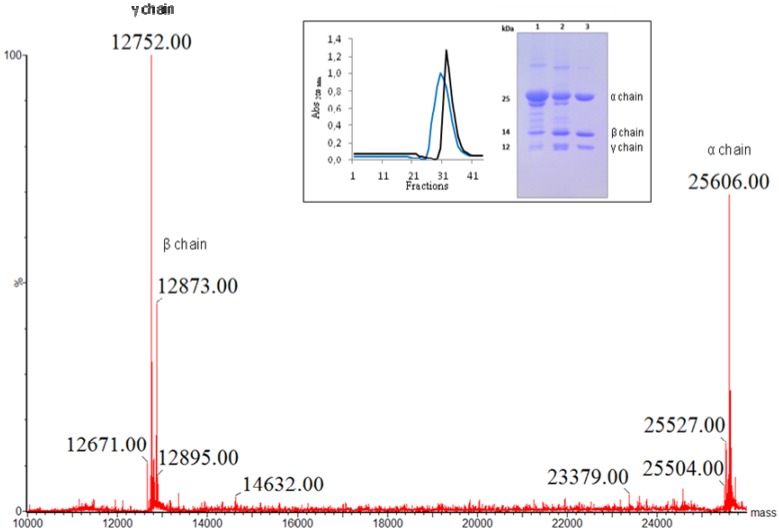
Deconvoluted mass spectra of *D. sclerocarpa* lectin showing the molecular masses of full length α-chain (25,606 Da) and its derived β- (12,832 Da) and γ- (12,752 Da) fragments. Inset: Purification of DSL by affinity chromatography on sephadex-G50. DSL eluted with 0.1 M glycine in 0.15 NaCl pH 2.6 (blue line) and with 0.1 M glucose in 0.15 M NaCl (black line). SDS-PAGE of DSL. 1 and 2—Lectin from *Canavalia brasiliensis *and *Dioclea violacea* seeds as molecular weight marker, respectively; 3—Lectin from *D. sclerocarpa *seeds.

DSL was easily purified in a single step by affinity chromatography on a Sephadex G-50 column. The unbound fraction displayed no lectin activity. The bound protein eluted with glucose had a specific activity of 2.09 × 10^6^ H.U./mg protein. The minimal concentration of puriﬁed protein required to agglutinate a 2% rabbit erythrocyte suspension was <4 × 10^−7^ mg. The purity and the apparent molecular weight of the lectin were determined by polyacrylamide gel electrophoresis after heating in the presence of SDS (insert, Figure 1).

DSL appears to be composed of three polypeptide chains weighing approximately 30, 14 and 12 kDa, matching the pattern of other Diocleinae lectins, such as *Dioclea violacea *[15] and *Dioclea grandiflora *[17]. These findings were confirmed by mass spectrometry analysis proving the existence of just one lectin, composed of three chains (α, β and γ). The α-chain has an exact molecular mass of 25,606 ± 2 Da. The mass of each fragment is 12,873 ± 2 Da (β-chain) and 12,752 ± 2 Da (γ-chain) (Figure 1).

The DSL exhibited a broad pH optimum (6.0–8.0), but lost 50% agglutination activity at pH 8.5. The incubation of DSL with EDTA resulted in loss of agglutination activity. Lost activity was only recovered by incubation with Ca^2+^ and Mn^2+^ at concentrations above 10 mM. The phenol sulphuric acid test detected no carbohydrate moiety in DSL.

**Figure 2:**
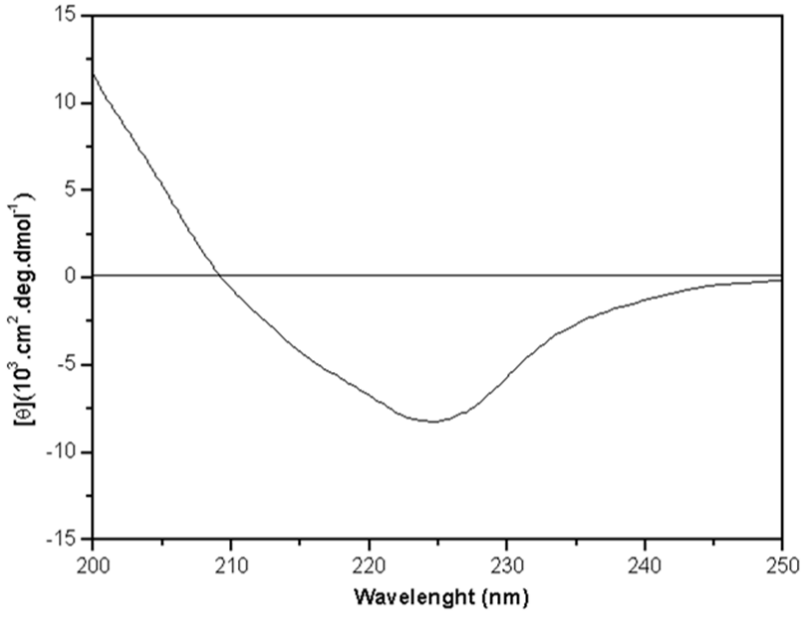
Far-UV circular dichroism spectrum of *D. sclerocarpa* lectin (DSL). The spectrum was measured from lectin at a concentration of 0.25 mg/mL in 20 mM phosphate buffer containing 10 mM NaCl, pH 8.0. Measurements were performed at 25 °C from 200 to 250 nm (average of 16 scans) using a rectangular quartz cuvette (1 mm path).

Figure 2 shows the CD spectrum of DSL in the natural state. The far-UV CD spectrum revealed a large negative peak at 224 nm, a positive band at 200 nm and a negative-to-positive crossover at 209 nm. The analysis of the secondary structure indicated that β-sheet secondary structures are predominant in DSL (approximately 42.3% antiparallel β-sheet and 6.7% parallel β-sheet). In addition to the β-sheet, the predicted secondary structure of DSL features 4.1% α-helices, 15.8% turns and 31.3% other contributions.

**Figure 3:**
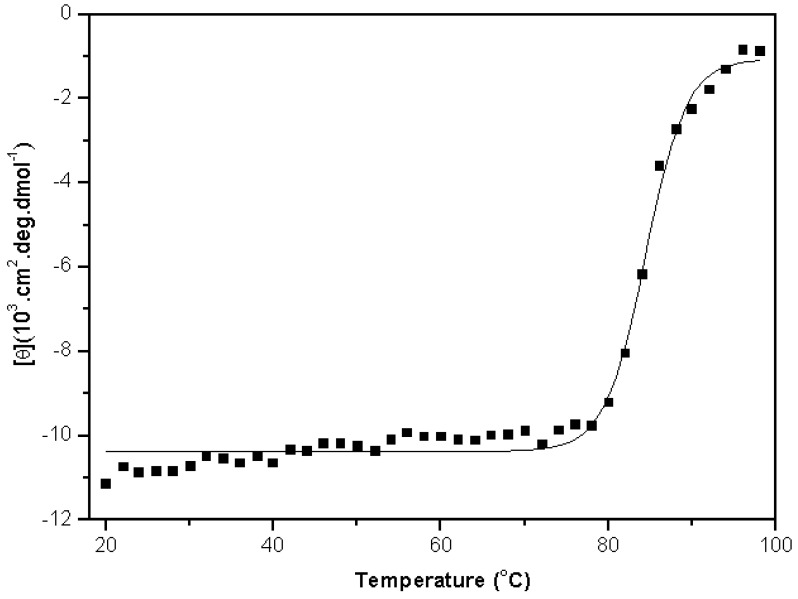
Thermal denaturation curve of native *D. sclerocarpa *lectin (DSL) monitored by Far-UV circular dichroism. Lectin denaturation was characterized by measuring changes in ellipticity at 218 nm by a temperature increase from 20 to 98 °C. DSL sample (0.25 mg/mL in 20 mM Tris-HCl buffer, pH 8.0) was heated gradually in steps of 2 °C. At each temperature, the protein was incubated for 1 min and the spectrum was recorded over a wavelength range of 200–250 nm (average of 16 scans). Measurements were performed using a rectangular quartz cuvette (1 mm path).

Upon thermal denaturation, DSL displayed cooperative sigmoidal behavior (Figure 3) with transition midpoint at 84 °C and permitted the observation of a two-state model (native and denatured). These results indicate that DSL is a heat resistant protein, similar to some other lectins of the legume family [7]. The thermal denaturation of DSL was found to be an irreversible process, since samples that were heated up to 98 °C did not return to their original state after either rapid or slow cooling back to 20 °C.

**Figure 4:**
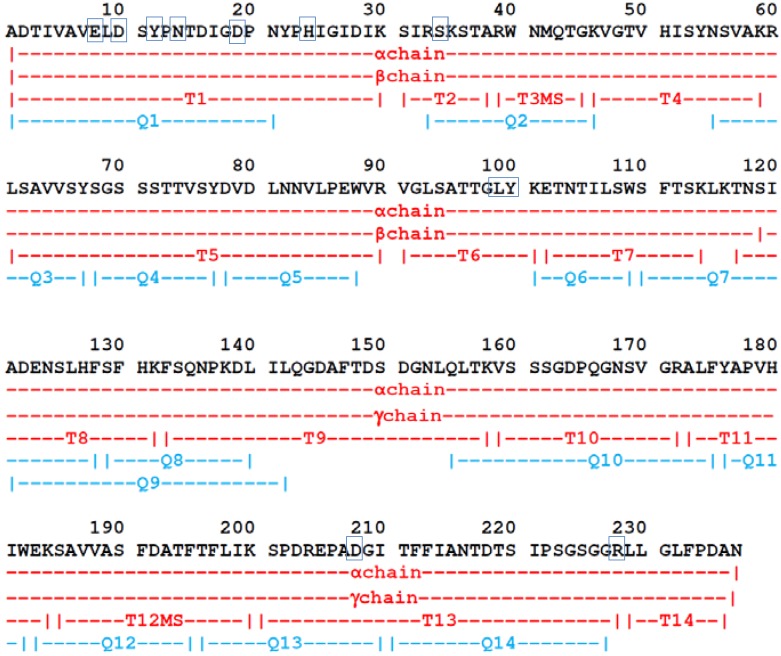
The amino acid sequence of *D. sclerocarpa* seed lectin, DSL. Peptides obtained by digestion of DSL with endoproteinases trypsin (T) and chymotrypsin (Q). The blue squares indicate conserved residues involved in metal and carbohydrate binding sites.

The amino acid sequence of DSL was established by tandem mass spectrometry analysis of sets of overlapping peptides obtained by proteolytic digestions. The primary sequence includes 237 amino acids distributed between the β-chain (residues 1-118) and the γ-chain (residues 119-237) (Figure 4, Table 1). The isotope-averaged molecular masses calculated for the full-length α-chain (25,606 Da) and its derived β- (12,873 Da) and γ- (12,753 Da) fragments are in excellent agreement with the experimentally determined mass.

**Table 1:** Sequenced peptides and their respective molecular mass.

Peptide	Experimental mass (Da)	Sequence
T1	3254.5981	ADTIVAVELDSYPNTDIGDPSYPHIGIDIK
T2	1004.5582	SIRSKSTAR
T3 *	863.3566	WNMQTGK
T4	1373.7257	VGTVHISYNSVAK
T5	3243.5935	LSAVVSYSGSSSTTVSYDVDLNNVLPEWVR
T6	1108.5526	VGLSATTGLYK
T7	1512.6473	ETNTILSWSFTSK
T8	1845.6696	TNSIADENSLHFSFHK
T9	4044.8477	FSQNPKDLILQGDAFTDSDGNLQLTK
T10	1345.6222	VSSSGDPQGNSVGR
T11	1472.7642	ALFYAPVHIWEK
T12 *	1715.8588	SAVVASFDATFTFLIK
T13	2864.1970	SPDREPADGITFFIANTDTSIPSGSGGR
T14	958.4971	LLGLFPDAN
Q1	2342.9988	DSYPNTDIGDPNYPHIGIDIK
Q2	1533.7388	NMQTGKVGTVHISY
Q3	1392.7252	NSVAKRLSAVVSY
Q4	974.4125	SGSSSTTVSY
Q5	1312.6182	DVDLNNVLPEW
Q6	1090.5066	KETNTILSW
Q7	2137.9294	SFTSKLKTNSIADENSLHF
Q8	1446.7222	SFHKFSQNPKDL
Q9	2246.9380	SQNPKDLILQGDAFTDSDGNL
Q10	1999.9325	QLTKVSSSGDPQGNSVGRALF
Q11	884.4222	YAPVHIW
Q12	1370.6240	KSAVVASFDATF
Q13	1544.6987	IKSPDREPADGITF
Q14	1804.9670	FIANTDTSIPSGSGGRLL

* Peptides identified by the peptide mass fingerprint (PMF). All the others peptides were sequenced by tandem mass spectrometry.

**Figure 5:**
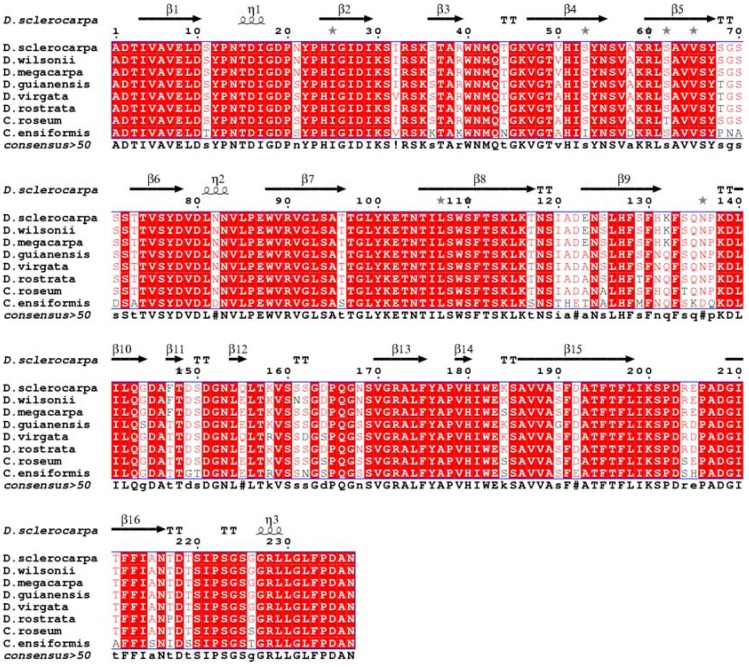
Sequence alignment among DSL and different Diocleinae lectins. Accession numbers: P86624 (*Dioclea wilsonii*), P08902 (*Dioclea megacarpa*), P81637 (*Dioclea guianensis*), P58907 (*Dioclea virgata*), P58908 (*Dioclea rostrata*), P86184 (*Cymbosema roseum)*, P50477 (*Canavalia ensiformis*), respectively.

Comparisons among DSL and other Dioclenae lectins through sequence alignment with Espript 2.1 [20] attested a high degree of sequence and structural homology (Figure 5). The highly conserved carbohydrate and metal-binding sites in DSL contain the same residues described for other ConA-like lectins [21] (Figure 4). The protein most similar to DSL is *D. megacarpa* (P08902) seed lectin, differing in only one amino acid at the position 155 (glutamine in the former, glutamic acid in the latter). The data obtained by CD are completely in accordance with the primary sequence data and the secondary structure prediction observed in Figure 5.

## 3. Experimental

### 3.1. Materials

Seeds of *Dioclea sclerocarpa* were harvested from plants growing in Ceará (Northeastern Brazil) and identified at the Department of Biology of the Federal University of Ceará (UFC). Erythrocytes were obtained from rabbits reared at the UFC research animal facility.

### 3.2. Protein Extraction Procedure

The seeds were ground to a fine powder using a coffee mill and the soluble proteins were extracted at room temperature in 0.1 M Tris-HCl buffer (pH 7.4) containing 0.15 M NaCl [1:10 (w:v)] under continuous stirring for 4 h, followed by filtration through filter paper (Whatman™) and centrifugation at 10,000 × *g* at 4 °C for 20 min. The supernatant was used for further experiments. The protein concentration was determined by the method described by Bradford [22] using bovine serum albumin (BSA) as standard.

### 3.3. Hemagglutinating Activity

Hemagglutinating activity (H.A) was determined using tubes with double serial dilutions [23]. All tubes received 0.1 M Tris–HCl buffer (pH 7.6, 100 μL) containing 0.15 M NaCl. An aliquot of protein extract (100 μL) was added to the first tube of the series. Subsequently, 2% rabbit erythrocytes suspension (100 μL) treated with trypsin, papain or neither, containing 0.15 M NaCl, was added to each tube. H.A was measured after 30 min of incubation at 37 °C and 30 min of incubation at room temperature. H.A was expressed as a titer (the reciprocal value of the highest dilution testing positive) per mg of protein.

### 3.4. Sugar Specificity

Sugar specificity was determined by comparing the ability of different sugars to inhibit DSL-induced H.A at four hemagglutination units. Using the methodology described above, diluted protein extract was incubated with sugars for 30 min at 37 °C prior to the determination of the inhibition titer. The initial concentration of the carbohydrates tested was 0.1 M. Results were expressed as the minimum concentration of sugar required to inhibit H.A.

### 3.5. Protein Purification

The supernatant obtained during protein extraction was applied onto a Sephadex G-50 column with 30 mL cross-linked dextran gel previously equilibrated with 0.15 M NaCl containing 0.005 M CaCl_2_ and 0.005 M MnCl_2_. The unbound material was washed with the same buffer at a constant flow rate until the absorbance of the effluent at 280 nm had reached the baseline. The retained fraction was eluted with 0.1 M glucose containing 0.15 M NaCl or 0.1 M glycine buffer (pH 2.6) until the absorbance of the effluent at 280 nm had been stabilized at 0.05 [24] and subsequently dialyzed against ultrapure water at room temperature for 12 h. The lectin (DSL) was then lyophilized for further analysis, including thermostability (CD experiments), different pH values, effect of EDTA on lectin activity and molecular mass determination by mass spectrometry. Lectin stability at different pH was determined after dialysis of the lectin solution (0.2 mg/mL in 0.15 M NaCl) against different pH (4.0 to 10.0) buffers containing 0.15 M NaCl for 24 h. The buffer solutions used in this experiment were 0.1 M sodium citrate (pH 4.0 and 6.0), 0.1 M sodium acetate (pH 5.0), 0.1 M sodium phosphate (pH 7.0), 0.1 M Tris-HCl (pH 8.0), and 0.1 M glycine-NaOH (pH 9.0 and 10.0).

### 3.6. Sodium Dodecyl Sulfate Polyacrylamide Gel Electrophoresis

Sodium dodecyl sulfate (SDS) polyacrylamide gel electrophoresis (PAGE) was performed using 0.75 mm vertical gel slabs with 15% polyacrylamide separation gel containing 0.375 M Tris-HCl (pH 8.8), 30% acrylamide/0.8% bis-acrylamide, 0.03% ammonium persulfate, tetramethylethylenediamine (TEMED) (pH 8.9) and 0.1% SDS buffer, and 4% stacking gel containg 0.25 M Tris-HCl (pH 6.8) with the same reagents than separation gel [25]. Samples were dissolved in 0.88 M Tris-HCl (pH 6.8), 2% SDS buffer, 5% β-mercaptoethanol, 1% bromophenol blue and 12.5% glycerol, followed by incubation at 100 °C for 5 min. Electrophoresis was conducted at a constant current of 25 mA for 90 min. The protein bands were visualized by staining with Coomassie Brilliant Blue R-250.

### 3.7. Lectin Metal Dependence

The metal dependence of the purified lectin (1 mg pure DSL dissolved in 1 mL pure water) was determined after 48 h of dialysis against 0.2 M EDTA, followed by dialysis against 0.15 M NaCl for 24 h. The dialyzed lectin solution was evaluated for H.A. Recovery of H.A was attempted by adding 0.005 M CaCl_2_ and 0.005 M MnCl_2_.

### 3.8. Carbohydrate Content Analysis

The carbohydrate content of the puriﬁed lectin was determined with the phenol sulphuric acid method as described by the Dubois method [26] using D-glucose as standard.

### 3.9. Circular Dichroism and Thermostability

Far-UV circular dichroism (CD) spectra were recorded on a JASCO J-815 spectropolarimeter (JASCO Corporation, Japan) equipped with a Peltier temperature control unit. The entire instrument, including the sample chamber, was constantly flushed with nitrogen gas during the operation. Measurements were performed using a rectangular quartz cuvette (1 mm path). Buffer scans were recorded under the same conditions and subtracted from the protein spectra before further analysis. CD spectra were the average of 16 accumulations at 25 °C, using a scanning speed of 50 nm/min. The DSL spectra were measured in protein solutions of 0.25 mg/mL in 0.02 M phosphate buffer containing 0.01 M NaCl, pH 8.0. The secondary structure analysis was performed by CD spectrum deconvolution using the SELCON3 [27]. For temperature stability studies, a protein sample (0.25 mg/mL in 0.02 M Tris-HCl buffer, pH 8.0) was heated gradually in 2 °C steps, from 20 to 98 °C. At each temperature step, the protein was incubated for 1 min and the spectrum was recorded over a wavelength range of 200–250 nm.

### 3.10. MW Determination by Mass Spectrometry

The molecular mass of DSL was determined by electrospray ionization using a hybrid mass spectrometer (Synapt HDMS system, Waters Corp., Milford, MA, USA). A protein solution (2.5 × 10^−4^ mg/mL) was infused into the system at a flow rate of 10 µL/min. The capillary voltage and the cone voltage were set at 3 kV and 40 V, respectively. The source temperature was maintained at 200 °C and nitrogen was used as a drying gas (flow rate of 150 L/h). The data were acquired with the software Mass Lynx 4.0. The multiply charged spectra were deconvoluted using maximum entropy techniques [28].

### 3.11. Protein Digestion and Tandem Mass Spectrometry Analysis

The SDS-PAGE bands were excised and bleached in a 0.05 M ammonium bicarbonate solution in 50% acetonitrile. The bands were dehydrated in 100% acetonitrile and dried in a Speedvac (LabConco). The gel was rehydrated with a 0.05 M ammonium bicarbonate solution containing trypsin (Promega) or chymotrypsin (Sigma) (1:50 w/w; enzyme: substrate ratio) at 37 °C overnight. Peptides were extracted from the gel, concentrated and injected into a nanoACQUITY system connected to the electrospray source of a mass spectrometer (SYNAPT HDMS system, Waters Corp.). The sample was applied to a C_18_ chromatography column (75 µm × 100 mm) and eluted with a 10–85% acetonitrile gradient containing 0.1% formic acid. The mass spectrometer operated in positive mode, using a source temperature of 90 °C and capillary voltage of 3.0 kV. The LC-MS/MS experiment was performed with the DDA (data-dependent acquisition) function selecting for MS/MS experiments with doubly or triply charged precursor ions fragmented by collision-induced dissociation. The data were processed and analyzed with a Proteinlynx v2.4 (Waters) using the peptide mass fingerprint (PMF) and the peptide fragmentation pattern as search parameters. Some peptide sequences were obtained by *de novo* sequencing.

### 3.12. Primary Structure Analysis

The primary sequence was submitted to BLAST analysis [29]. The proteins with the best e-value were selected for Multalin alignment [30]. The alignment with secondary structure prediction was made with the software ESPript 2.2 [20]. The theoretical MW and pI were calculated with the software ProtParam [31].

## 4. Conclusions

DSL is a glucose/mannose-binding lectin purified from the protein extract of the seeds of *Dioclea sclerocarpa*. Like other Diocleinae lectins, DSL is a monomer with molecular mass of 25,606 Da, which associates as a tetramer. Its mature α-chain is produce through a post-translational circular permutation—A typical Diocleinae post translation modification—cleaving the pre-pro-protein in two small chains (β and γ). The α-chain polypeptide has 237 amino acids and is classified as a Con-A like lectin based on its primary amino acid sequence. 

*D. sclerocarpa* and *D. megacarpa* only differ by one residue (155) in the mature chain. Therefore, lectins can be used as metabolic markers to differentiate legume species that share morphological similarities. Further investigations about the biological properties of DSL may provide important clues to determine whether DSL, like other legume lectins, present potential for technological applications.
